# Image Statistics Preserving Encrypt-then-Compress Scheme Dedicated for JPEG Compression Standard

**DOI:** 10.3390/e23040421

**Published:** 2021-03-31

**Authors:** Dariusz Puchala, Kamil Stokfiszewski, Mykhaylo Yatsymirskyy

**Affiliations:** Institute of Information Technology, Lodz University of Technology, 90-924 Lodz, Poland; kamil.stokfiszewski@p.lodz.pl (K.S.); mykhaylo.yatsymirskyy@p.lodz.pl (M.Y.)

**Keywords:** encryption of images, compression of images, linear discrete parametric transforms, transform coding, uniform block quantization, entropy coding

## Abstract

In this paper, the authors analyze in more details an image encryption scheme, proposed by the authors in their earlier work, which preserves input image statistics and can be used in connection with the JPEG compression standard. The image encryption process takes advantage of fast linear transforms parametrized with private keys and is carried out prior to the compression stage in a way that does not alter those statistical characteristics of the input image that are crucial from the point of view of the subsequent compression. This feature makes the encryption process transparent to the compression stage and enables the JPEG algorithm to maintain its full compression capabilities even though it operates on the encrypted image data. The main advantage of the considered approach is the fact that the JPEG algorithm can be used without any modifications as a part of the encrypt-then-compress image processing framework. The paper includes a detailed mathematical model of the examined scheme allowing for theoretical analysis of the impact of the image encryption step on the effectiveness of the compression process. The combinatorial and statistical analysis of the encryption process is also included and it allows to evaluate its cryptographic strength. In addition, the paper considers several practical use-case scenarios with different characteristics of the compression and encryption stages. The final part of the paper contains the additional results of the experimental studies regarding general effectiveness of the presented scheme. The results show that for a wide range of compression ratios the considered scheme performs comparably to the JPEG algorithm alone, that is, without the encryption stage, in terms of the quality measures of reconstructed images. Moreover, the results of statistical analysis as well as those obtained with generally approved quality measures of image cryptographic systems, prove high strength and efficiency of the scheme’s encryption stage.

## 1. Introduction

The beginning of the twenty-first century brings the dynamic development of telecommunication technologies giving the possibility of practical use of multimedia in almost all areas of our lives. The successively increased bandwidths of data transmission channels enable fast transmission of high resolution images and video sequences. This allows for the wide spread of remote data exchange systems for audio/video conferencing in real time, publishing multimedia content in computer networks through websites or image data exchange between experts in various fields using dedicated database systems, that is, in medical sciences, engineering or forensics.

The transmission of multimedia data using publicly available open communication channels makes the data vulnerable to interception and overhearing by unauthorized parties. This problem can be solved with cryptographic algorithms. The security of multimedia data transmission can be ensured by conventional cryptographic algorithms, that is, block ciphers such as Data Encryption Standard (DES) [1], International Data Encryption Algorithm (IDEA) [2], or Advanced Encryption Standard (AES) [3]. However, the systems based on encryption of entire multimedia data streams with block ciphers are referred to in the literature as *naïve approaches* (see [4]) because of their high computational complexity, lack of data stream consistency in video transmissions or incompatibility of encrypted data stream with devices that do not operate with the proper (or any) private key. In practice, selective approaches are preferred, that is, approaches where only the selected elements of the multimedia data stream are encrypted.

Both naïve and selective approaches can only be applied to *compress-then-encrypt* (CTE) systems, in which encryption is done after compression, or is combined within a given system with the compression method. The conversely defined problem, which requires *encrypt-then-compress* (ETC) approach and puts much higher demands on the cascade combination of encryption and compression methods, was formulated and addressed in papers [5,6,7,8,9,10,11]. In the ETC approach, the encryption step proceeds independently of the compression, and what is more important, the data is encrypted in the first place and then compressed in the following step. It should be emphasized that both steps are strongly contradictory. The aim of the encryption process is to hide all similarities (correlations) in the image. In turn, the compression process allows for the reduction of the data size based on the similarities and resulting redundancy of the representation.

**Figure 1:**
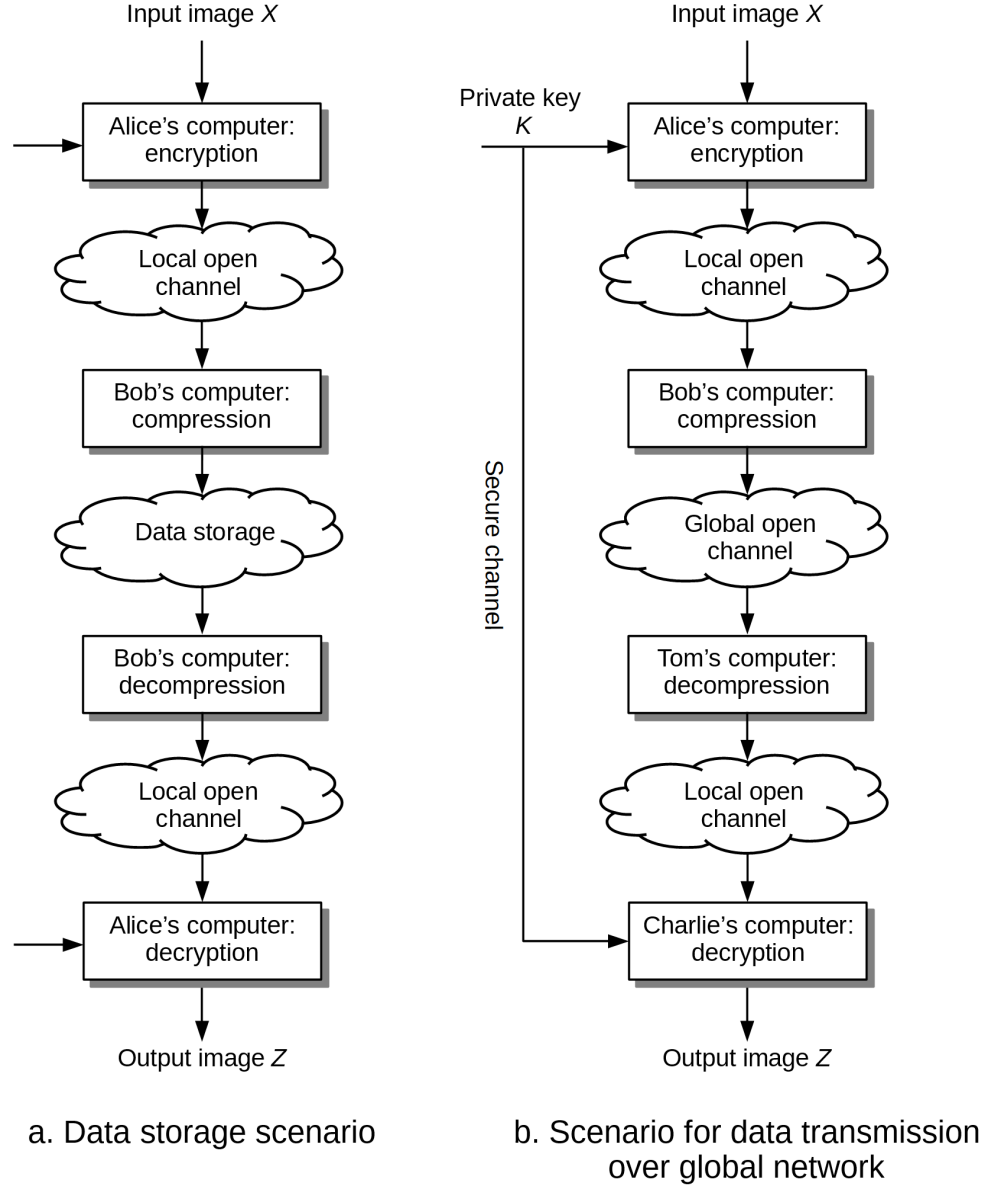
Exemplary scenarios for data encryption and compression according to encrypt-then-compress (ETC) scheme: data storage (**a**), data transmission over global network (**b**).

The ETC coding scenarios considered in this paper, which concern the common problems of data storage or data transmission over global network, are depicted in Figure 1. Both scenarios involve two actors—Alice and Bob, each with distinct needs and limited mutual trust. We assume that Alice wants to send confidential image data over the network or store it in an external archive. Both tasks are delegated to Bob, however he’s either not authorized to access the data or the data is sent to him over an open channel. For this reason Alice performs the data encryption process, being the only activity she’s forced to undertake in the assumed circumstances. Bob on the other hand, since his task is to deposit the data or forward it over the network, is highly interested in its compression, thus reducing the requirements for the available free disk space or the bandwidths of data transmission channels. In the ideal case Alice sends the ciphertext to Bob, who is able to compress it efficiently without any additional information, using known standards and tools. Such assumptions put even higher demands on the solutions being developed.

The scheme addressed in this paper is an example of such a solution that tries to meet all the requirements specified above.

In this paper we analyze in more details the novel scheme for encryption and compression of images according to the ETC scenario, which was was proposed during the 2020 Data Compression Conference (see [12]). The encryption stage takes advantage of linear orthogonal transforms and the known approach of image data mixing within blocks selected from different areas of an image [13]. Such operation in the encryption process allows to keep the statistical characteristics of the image, which is crucial from the point of view of the succeeding compression stage. The compression stage adopts a classical method based on scalar quantization in the domain of linear transformation (in practice *Discrete Cosine Transform* (DCT)) and entropy coding. The practical implementation of such method is the well known JPEG standard for lossy image compression. The analyzed scheme allows for the practical realization of the ETC scenario with the use of standard compression tools like JPEG, while maintaining the efficiency of compression at a level comparable to a case without the encryption stage.

The elements of novelty in this paper include: (i) the extended theoretical efficiency analysis of the scheme’s compression process along with additional, practical verification of its effectiveness, (ii) the proposal of utilization of fast parametric transforms as the effective computational tools which can be used in the encryption process, (iii) both the detailed theoretical and practical analysis of the efficiency of encryption stage.

## 2. Review of Existing Solutions

The ETC schemes proposed in the literature are based on a variety of approaches, that is, source coding with additional information [5,6,7], iterative image reconstruction based on reduced representation in the orthogonal transform domain [8], encryption and compression in the domain of integer wavelet transform [9], compressive sampling [10], the elements of game theory [11]. Depending on the adopted approach, we can expect different values of the compression ratio CR obtained at different values of the *Peak Signal-to-Noise Ratio* (PSNR), as well as different levels of compliance with the considered scenarios of practical usage (see Figure 1). In particular, we have approaches that exploit:

- *Source coding with additional information*: compression is based on known statistical relationships between ciphertext and the private key. If the elements of the ciphertext and the private key are correlated to the extent that the Hamming distance between them can be bounded from above, that is, the distance is not greater than ξ, then this information can be effectively used by the compression algorithm to reduce the size of the data without knowing the private key. It is enough to divide the set of values of ciphertext elements into layers in which we place those elements that are distant by more than 2ξ bits, whereas to the output stream we write not the values of elements themselves, but the identifiers of the layers to which they belong. In paper [5] it was shown that under certain conditions it is possible to compress encrypted data to the same level as in the case of compression of original data, that is, not subjected to encryption. The Authors also proposed a scheme for the encryption and lossless compression of binary images using LDPC (*Low-Density Parity Check Codes*) correction codes and XOR operation at the compression and encryption stages respectively. It allowed to obtain the practical compression ratios at the level of CR=1.3. In paper [6], the original approach was improved by taking into account the spatial dependencies between the values of neighboring pixels in the image. This allowed to increase the value of the compression ratio to the level of CR=2.3. Another improvement of the original method for grayscale and color images was proposed in paper [7]. The average values of compression ratios were obtained at the level of CR=1.8. The essential drawbacks of approaches based on source coding are relatively low levels of the compression ratio, and the lack of symmetry of the entire scheme, which requires to combine decompression and decryption stages. In practice, it means that such approaches do not fully follow the scenarios depicted in Figure 1.
- *Approximate representation of image pixels in the domain of linear transform*: such approach was proposed in paper [8], here at the encryption stage the image pixels are scrambled with use of permutation determined on the basis of the private key, whereas the compression stage assumes to divide the elements of ciphertext into two sets: (a) rigid pixels that are not further modified, (b) flexible pixels. The values of flexible pixels are represented in the domain of linear orthogonal transform and then quantized, while the results are assigned to a specific equivalence classes resulting from the division operation. It allows to describe the value of each of the obtained coefficients in the form of a weighted sum of three components: (a) coarse, which can be estimated based on the values of the nearest rigid pixels, (b) the average, which next to the values of rigid pixels is written to the output stream, (c) detailed, which is rejected. The values of rigid pixels and representations of transform coefficients that describe the components of the average elastic pixels are written to the output stream. The compression itself is lossy. The reconstruction of the image is possible based on the proposed iterative procedure. The practical values of the compression ratio obtained with this method are at the level of CR=3 with PSNR values around 35 dB.
- *Image representation in the domain of discrete integer wavelet transform (DIWT)* (see [9])—at the encryption stage, the grayscale input image is transformed using DIWT into one coarse band and nine bands containing detailed information. The data contained in the coarse band is encrypted by adding to it a sequence of pseudo-random numbers, while the range of resulting values is limited by the modulo division operation. The data contained in the remaining bands is permuted. Both the the pseudo-random sequence and the permutation are determined on the basis of the private key. The compression stage operates only on the encrypted data coming from the detailed bands. The data taken from those bands is quantized and then entropy coded using arithmetic coding. The compression and encryption stages are reversible, with the whole scheme being symmetrical. The practical results obtained with this method are very close to those obtained with JPEG standard. It should be emphasized that the compression method used here is a lossy one. Hence it is possible to obtain high compression ratios around CR=20 at the expense of quality distortion, but still with PSNR above 30 dB.
- *Compressive sensing* (see [7])—at the encryption stage, the image is reshaped into a single vector, and then encrypted by a linear method consisting of multiplying the input vector by any matrix, for example, permutation matrix, which is generated on the basis of a private key. The compression stage is based on compressive sampling and relies on projecting the ciphertext vector onto a set of basis vectors from the subspace with reduced size. Such a basis is most often a set of linearly-independent vectors whose elements are randomized. In addition, the coefficients of projections are quantized. In this way the reduction in the size of the data can be obtained. The image decoding process is based on the theory of compression sensing, wherein the matrix of discrete cosine transform (DCT) is taken as a matrix allowing for a sparse representation of the input image. During the experiments the practical values of the compression ratios were at the level of CR=2 with the PSNR coefficients around 30 dB. However, the discussed scheme is asymmetrical, which manifests in the way that the decompression and decryption stages must be combined.
- *Game theory*—proposed in paper [11], it is an improvement of the previously described approach from paper [8]. Here, at the encryption stage the image is divided into blocks of arbitrary sizes (e.g., 32×32 pixels), the order of which, the same as the order of pixels within the blocks, is modified using permutations described by the private key. An additional action is to determine the block type, that is, whether it is a texture or a smooth part of an image. Its aim is to increase the efficiency of the compression step. However, such actions must be done by the sender, which is surely a disadvantage of this approach. The compression step is based on the [8] approach, wherein the algorithm is applied to subsequent blocks, not to the whole image. Then, depending on the type of block, the value of a coefficient describing the share of rigid and elastic pixels, as well as the quantization step can be selected individually for each block. The choice of parameter values is adaptive and controlled by an algorithm based on the game theory, where image quality is being maximized while keeping the limit on the size of image after compression. Image reconstruction is based on the iterative technique proposed in [8]. During the experimental research, the quality of smooth images and textures was at the level of 36 and 27 dB, with the compression ratios around CR=2.32, which is an improvement of about 3 and 1 dB respectively when compared to the original approach from paper [8].

## 3. Mathematical Model of the Analyzed Scheme

In this section, we will present the mathematical model of the proposed scheme, and show the main characteristics of the compression and encryption processes present within its course.

**Figure 2:**
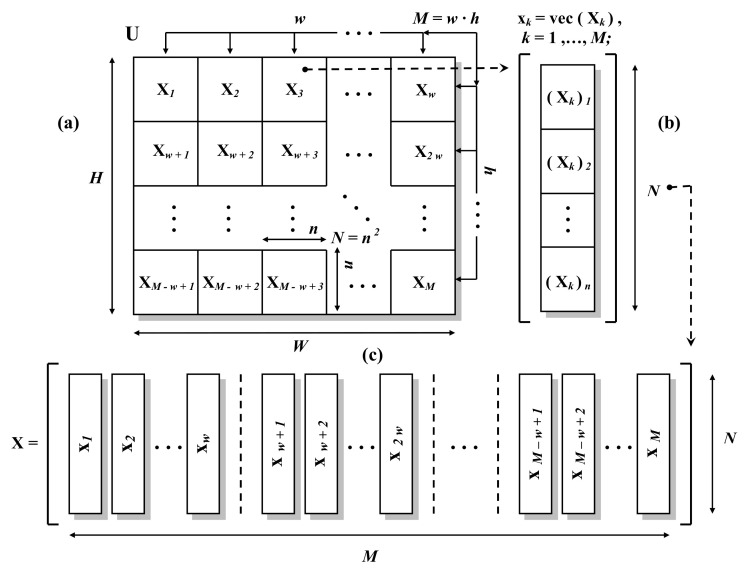
The analyzed scheme’s input data preparation.

Let us assume that the input of the considered scheme is a monochromatic image being a realization of some two-dimensional, stationary, zero-mean random field Ψ. Let w,h,n∈N and W=w×n, H=h×n and let’s suppose that M=w×h and $N=n^{2}$. In such case the image will be represented by H×W element matrix U, with elements $u_{ij}\in R$ for i=1,…,H and j=1,…,W. At first, initial arrangement the image’s input data is performed, what is shown in Figure 2.

In step (a), the input image U is divided into separate, square fragments $X_{k}$, k=1,…,M, each of which being the n×n element real matrix. Input image’s matrix U components mapping to the elements of matrices $X_{k}$ can be compactly written in the form of the following relationship: ∀k∈1,…,M and ∀i,j∈1,…,n:

(1) $${\left(\;X_{k}\right)}_{ij}=u_{\;\lfloor (k-1)/w\rfloor \times n\;+\;i,\;\;((k-1)\;\;mod\;\;w)\times n\;+\;j}\;,\;\;\;\;$$

where the symbol ⌊×⌋ stands for the *floor* function and *mod* denotes the integer modulo operation. In the next step, that is, step (b) in Figure 2, each of the $X_{k}$ matrices is flattened, creating the respective $N=n^{2}$ – element vector $x_{k}$ of the form:

(2) $$x_{k}=vec\;\left(\;X_{k}\right)={\left[\;\;{\left(\;X_{k}\right)}_{1}^{T}\;\;\;{\left(\;X_{k}\right)}_{2}^{T}\;\;\ldots \;\;{\left(\;X_{k}\right)}_{n}^{T}\;\;\right]}^{\;T},$$

where ${\left(\;X_{k}\right)}_{l}$, k=1,…,M, l=1,…,n, is the *l*-th column of the matrix $X_{k}$ and vec(×) is the matrix column vectorization operator, see for example [14]. In the last step, that is, step (c), *N* – element vectors $x_{k}$ are arranged into successive columns of the N×M – element matrix X, that is:

(3) $$\forall \;k\in \left\{\;1,\ldots ,M\;\right\}\;\;\;{\left(\;X\;\right)}_{k}=x_{k}\;,$$

where ${\left(\;X\;\right)}_{k}\;$ is the *k*-th column of the direct input matrix X, which constitutes the final form of the input data arrangement in the considered coding scheme.

**Figure 3:**
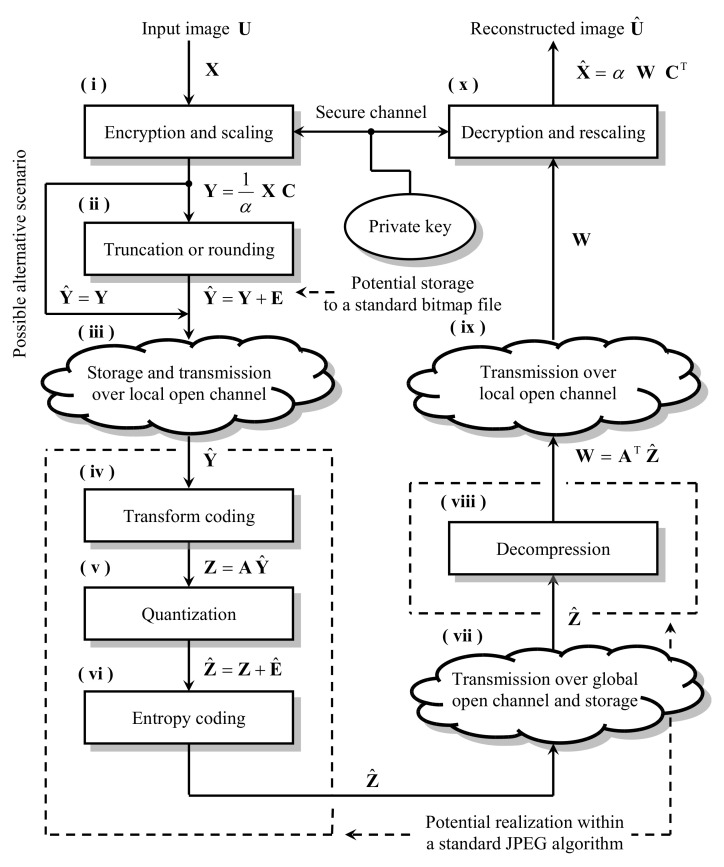
The course of the examined encryption and compression scheme.

After the input matrix X has been prepared, the coding process begins, whose course is schematically shown in Figure 3.

In the first step, denoted by (i), the input matrix X is encrypted and scaled, that is, Y=1/αXC, where C is the M×M – element, real, orthogonal, that is, $CC^{T}=I$, encryption matrix and α∈R is the scaling factor, whose value will usually be greater than 1, ensuring the range of the magnitudes of the elements of the ciphertext XC being acceptable from the point of view of the subsequent coding stages. Step (ii) is optional and, for specific implementations of the analyzed scheme, might involve acquisition of the scaled ciphertext Y in the form of the preferred standard graphics file format, for example, BMP or PNG (see e.g., [15]). This enforces truncation or rounding of the ciphertext data to the respective integer values, since most of the graphics file formats assume their input image data to be coded as integers. This is modeled by adding the N×M – element integer projection error matrix E to the ciphertext Y (see e.g., [16,17]), resulting in the integer-valued matrix $\hat{Y}$. Steps (iv), (v) and (vi) comprise the actual image compression process which follows exactly the JPEG image compression algorithm’s operation (see [18]). In those steps the JPEG method can be utilized without any modifications. In step (iv) the ciphertext $\hat{Y}$ is transformed by the orthogonal, N×N – element compression matrix A, then it is quantized and entropy coded. Quantization is modeled by adding the N×M – element rounding error matrix $\hat{E}$ to the matrix Z, resulting in the integer-valued matrix $\hat{Z}$. In step (vii) the compressed ciphertext is sent through the open communication channel to the destination device. Decompression, which is performed in step (viii), is the exact reverse of the compression process taking place in steps (iv), (v) and (vi). At this stage we assume the decompression matrix $A^{T}$ to be the inverse of the orthogonal compression matrix A applied in step (iv). Here, the unmodified JPEG algorithm can also be fully utilized, resulting in the obtainment of the N×M – element, real matrix W. In the last stage (x) of the considered scheme, the decryption and rescaling of the values of the matrix W is carried out, what results in obtaining the N×M – element matrix $\hat{X}$, which approximates the input image matrix X. The analyzed encryption before compression coding scheme, described above, can be stated in terms of the following model equation:

(4) $$\hat{X}\;=\;\alpha \;\;A^{T}\;\left(\;\;A\;\;\left(\;\;\alpha^{-1}\;X\;C\;+\;E\;\;\right)\;+\;\hat{E}\;\;\right)\;\;C^{\;T}.$$

Equation (4) comprises the mathematical model of the examined scheme and is used as the basis for derivation of its most significant efficiency characteristics.

## 4. Compression Process Effectiveness Analysis

In this section we will present and analyze the equations describing the efficiency of the compression process present within the considered image coding scheme. For this purpose, on the basis of the model Equation (4), using high-resolution approximations to Shannon’s information theory, for example, [19,20,21,22,23], along with the results developed in our previous work [12], we’ll derive the *distortion-rate* characteristics of the analyzed scheme, that is, the D(R) function, whose explicit form comprises the exhaustive description of the effectiveness of the analyzed image compression process. Eventually, we’ll show that: (i) the obtained D(R) function does not depend on the choice of the encryption matrix C, and (ii) the encryption step preserves the second order statistics of the input image data. Both mentioned features are the main characteristics of the image coding scheme examined in this work.

We will solely base our analysis on the results obtained in our previous work (please refer to [12] for all the detailed derivations), in which it is initially stated that the mean squared error of reconstruction of the input signal at the output of the considered scheme takes the following form:

(5) $$D=\frac{1}{MN}\;\;tr\;\left\{\;\left(\;\hat{X}-X\;\right)\;{\left(\;\hat{X}-X\;\right)}^{T}\right\}\;,$$

where tr{×} is the matrix trace operator. Using orthogonality of the compression and encryption matrices A and C, respectively, we can simplify the form of the image $\hat{X}$, reconstructed at the output of the analyzed scheme, obtaining:

(6) $$\hat{X}\;=\;X\;+\;\alpha \;\left(\;A^{T}\;\hat{E}\;+\;E\;\right)\;C^{\;T}\;.$$

Substituting Equation (6) to the relationship (5) and using proper sample approximations, $\bar{E}$ and $\bar{\hat{E}}$, of the integer projection and quantization errors’ matrices, respectively (see [12]), present in steps (ii) and (v) of the examined scheme (c.f. Figure 3), we conclude that:

(7) $$D=\alpha^{2}\;\frac{1}{N}\;\left(\;\sum_{i=1}^{N}\sigma_{e_{i}}^{2}\;+\;\sum_{i=1}^{N}\sigma_{{\hat{e}}_{i}}^{2}\;\right)\;,$$

where α is the scaling factor used in steps (i) and (x) of the analyzed scheme, while $\sigma_{e_{i}}^{2}$ and $\sigma_{{\hat{e}}_{i}}^{2}$ are the sample estimators of integer projection and quantization errors’ variances (see [12]). It is worth explaining here, that in case of the optional scenario (c.f. step (ii) in Figure 3), in which the user wishes to archive the encrypted image XC in the form of one of the selected standard graphics file formats, e.g BMP, the concrete value of the scaling factor α, proper for the particular input image X, has to be chosen in such a way, that all integer projected values $\frac{1}{\alpha }\;X\;C+E$ of the scaled ciphertext $\frac{1}{\alpha }\;X\;C$ must fall into an interval contained within the appropriate input range, accepted by that selected format, for example, in case of 8-bit grayscale BMP format images, $\frac{1}{\alpha }\;X\;C+E$ must fall (after 128 level-shift) into an interval [0,…,255]⊂Z. The detailed discussion on the values of the scaling factor α, proper for our model’s assumptions, will be carried out in the next section.

**Figure 4:**
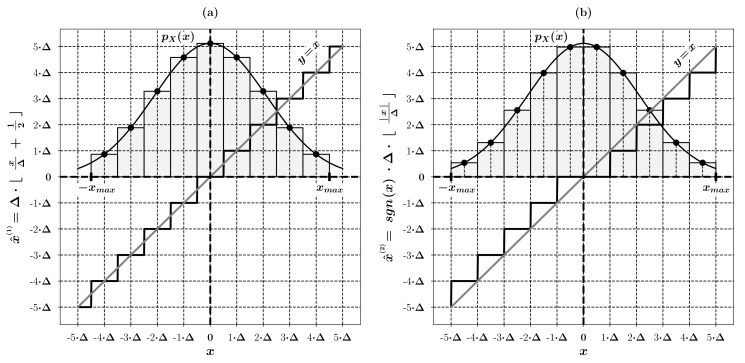
Schematic view of the operation of quantizers (8) for an exemplary random variable *X*.

Let us now examine the problem of approximation of the sample estimators of integer projection and quantization errors’ variances, that is, parameters $\sigma_{e_{i}}^{2}$ and $\sigma_{{\hat{e}}_{i}}^{2}$, i=1,…,N present in the Equation (7), which result from performing the steps (ii) and (v) within the examined image coding scheme. Let’s consider uniform scalar quantization of a continuous, one-dimensional random variable *X* with sufficiently smooth probability density function $p_{X}$, performed with two types of scalar quantizers, ${\hat{x}}^{\left(1\right)}$ and ${\hat{x}}^{\left(2\right)}$, whose reconstruction levels for an arbitrary value x∈R of a random variable *X* are given by the following relationships:

(8) $${\hat{x}}^{\left(1\right)}\left(x\right)=\Delta \times \left\lfloor \;\frac{x}{\Delta }+\frac{1}{2}\;\right\rfloor \;\;\;\;\;\mathrm{and}\;\;\;\;\;{\hat{x}}^{\left(2\right)}\left(x\right)=sgn\left(x\right)\times \Delta \times \left\lfloor \;\frac{\left|\;x\;\right|}{\Delta }\;\right\rfloor \;,$$

where $\Delta \in R^{+}$ denotes the quantization step. The operation of both of the considered quantizers is depicted schematically below in Figure 4.

Quantizers ${\hat{x}}^{\left(1\right)}$ and ${\hat{x}}^{\left(2\right)}$ perform rounding and truncation, respectively, of the variable’s *X* values to the nearest multiplicities of their quantization steps. With such assumptions we can write the common expression for the quantization error variance $\sigma_{e^{\left(k\right)}}^{2}$, for both of the considered quantizers ${\hat{x}}^{\left(k\right)}$, k=1,2, in the following way:

(9) $$\sigma_{e^{\left(k\right)}}^{2}=\sum_{i\;=\;1}^{\;L\;}\;\int_{x_{i-1}}^{x_{i}}{\left(\;\;x-{\hat{x}}^{\left(k\right)}\left(x\right)\right)}^{\;2}\;p_{X}\left(x\right)\;\;dx\;,$$

where N∋L≫1 denotes the number of quantization levels of a selected quantizer and $x_{j}=-\frac{1}{2}\;L\;\Delta +j\;\Delta$, j=0,…,L, are the limits of its consecutive reconstruction levels’ intervals $[\;x_{i-1},x_{i}\;]\subset R$, i=1,…,L. Taking advantage of the well-known results of high resolution quantization theory, see [20,21,22], we can infer that the expression (9) can be approximated by the following relationships:

(10) $$\sigma_{e^{\left(1\right)}}^{2}=\frac{\;\Delta^{2}}{12}\;\;\;\;\;\mathrm{and}\;\;\;\;\;\sigma_{e^{\left(2\right)}}^{2}=\frac{\;\Delta^{2}}{3}\;,$$

appropriate for the considered rounding ${\hat{x}}^{\left(1\right)}$ and truncation ${\hat{x}}^{\left(2\right)}$ quantizers, respectively.

Moreover, setting Δ≡1 in Equation (10), lets us approximate the error variances for the integer rounding and integer truncation towards zero operations, which eventually take the following forms:

(11) $$\sigma_{e}^{2}\;=\;\left\{\begin{array}{cc} \;\;\;\frac{1}{12} & \mathrm{for}\;\mathrm{rounding},\\ \;\;\;\;\frac{1}{3} & \mathrm{for}\;\mathrm{truncation}. \end{array}\right.\;$$

Going back to the main course of our considerations and using the results stated in Equations (10) and (11), we can rewrite the expression (7) for the mean squared error *D* of the reconstruction of the input image at the output of the analyzed scheme, as follows:

(12) $$D\;≅\;\alpha^{2}\;\frac{1}{N}\;\sum_{i=1}^{N}\;\frac{\;\Delta_{i}^{2}}{12}\;+\;\alpha^{2}\;\sigma_{e}^{2}\;,$$

where $\Delta_{i}^{2},\;\;i=1,\ldots ,N$ are the steps of the independent scalar quantizers used in stage (v) of the examined scheme and $\sigma_{e}^{2}$ can take alternative values given in (11), depending on the chosen integer projection operation applied in step (ii) of the analyzed scheme.

Let us now concentrate the on the evaluation of minimum average bit rate *R*, being the bit rate in the sense of the Shannon’s *rate-distortion theory* [19], of representation of a single sample coded at the output of the examined scheme, corresponding to the mean squared error *D*, given in (12). On the basis of the detailed derivation presented in our earlier work [12], we can immediately state that the approximate value of the considered bit rate may be expressed as:

(13) $$R≅\frac{1}{2}\;log_{\;\;2}\left(\;\frac{2\pi e}{\;\alpha^{2}}{\left(\;\prod_{\;i=1}^{N}\;\frac{a_{\;i}^{T}\;R_{x}\;a_{\;i}\;+\;\alpha^{2}\sigma_{e}^{2}}{\Delta_{i}^{2}}\;\;\right)}^{\frac{1}{N}}\right)\;,$$

where $a_{\;i}^{T}$, i=1,…,N is the *i*-th row of the orthogonal compression matrix A, utilized in step (iv) of the considered scheme, and $R_{x}=1/M\;X\;X^{T}$ is the sample autocovariance matrix of the input image X. Using dependencies (12) and (13), after some mathematical manipulations (once again please refer to [12] for the details), we obtain the explicit form of the relationship between the analyzed mean squared error *D* and its corresponding minimum average bit rate *R*, characteristic of the analyzed coding scheme:

(14) $$D\;\left(\;R\;\right)\;≅\;\frac{{\pi e\;∥\Delta ∥}^{2}}{6N}\;{\left(\;\prod_{\;i=1}^{N}\;\frac{a_{\;i}^{T}\;R_{x}\;a_{\;i}\;+\;\alpha^{2}\sigma_{e}^{2}}{\Delta_{i}^{2}}\;\right)}^{\frac{1}{N}}\times \;2^{-2R}\;+\;\alpha^{2}\sigma_{e}^{2}\;,$$

where ∥Δ∥ is the Euclidean norm of $\Delta =[\;\Delta_{1},\Delta_{2},\ldots ,\Delta_{N}\;]$, that is, the vector of the quantization table coefficients. According to Shannon’s information theory, the relationship between the measures *D* and *R*, given by the approximate dependency (14), that is, the *distortion-rate function*, is an exhaustive description of the effectiveness of image compression process being the part of the coding scheme considered in this paper.

By analyzing Equation (14), describing the distortion-rate function for the examined coding scheme, one can conclude that *the effectiveness of the compression process does not depend on the choice of the encryption matrix* C. Moreover, we have:

$$R_{y}=\frac{1}{M}\;Y\;Y^{T}=\frac{1}{\alpha^{2}M}\;X\;C\;\;C^{T}X^{T}=\frac{1}{\alpha^{2}M}\;X\;X^{T}=\frac{1}{\;\alpha^{2}}\;R_{x},$$

therefore, *from statistical point of view of the compression process, which follows the input image’s encryption step, both signals, input* X *and encrypted* Y, *are equivalent*, up to a scaling factor, what is compensated in the last step of the analyzed scheme. Both mentioned features are the main characteristics of the presented image coding scheme and stood originally at the basis of its construction.

### Examples of Compression Process Quality Characteristics

In this part of the work we will present examples of theoretical image compression process quality characteristics, achieved by the examined image coding scheme under selected operational scenarios, along with the exemplary practical results allowing for brief verification of the accuracy of the derived approximation of the distortion-rate function (14), characteristic to the considered scheme.

Let us assume a global image model (see [24,25]), in which the image is considered to be the realization of some two-dimensional, discrete index, zero-mean, stationary, separable random field with $W^{\;2}\times W^{\;2}$ – element autocovariance matrix of the form:

(15) $$R_{x}=E\;\left\{\;\;vec\;\;\left(\;\;U\;\;\right)\;\;vec\;\;\left(\;\;U^{T}\right)\;\right\}\;=\;\sigma_{x}^{2}\;\;R\;\left(\;\rho_{r}\right)\;⊗\;R\;\left(\;\rho_{c}\right),$$

where U is W×W – element stochastic matrix representing the image itself, E{×} is the expected value operator, ⊗ is a matrix Kronecker product (see e.g., [14]), $\sigma_{x}^{2}$ is the variance of a single random variable of the field U, $\rho_{r},\rho_{c}\in \left(-1,1\;\right)$ are row and column correlation coefficients, respectively, of adjacent elements of the U matrix, and the individual elements of the W×W – element matrix R(ρ) are defined as follows:

(16) $${\left[\;\;R\;\left(\;\rho \;\right)\;\right]}_{\;ij}=\rho^{\;|i-j|}\;,\;i,j=1,\ldots ,W,\;\rho \in \left(-1,1\;\right).$$

Let us assume further that the M×M – element, orthogonal encryption matrix C has the following form:

(17) $$C=P_{r}\;diag\;\left(\;C_{1},C_{2},\ldots ,C_{M/K}\;\right)\;P_{c}\;,$$

where $diag\;(\;C_{1},\ldots ,C_{M/K})\;$ is a block-diagonal, orthogonal real matrix, composed of K×K – element orthogonal matrices $C_{i}$, i=1,…,M/K, while additional M×M element permutation matrices $P_{r}$ and $P_{c}$ apply respective permutations to the rows and to the columns of the block-diagonal matrix $diag\;(\;C_{1},\ldots ,C_{M/K})\;$. Such choice of the form of the encryption matrix C is very useful practically since it enables, for example, the calculation of the ciphertext image with the use of fast parametric transformations (see Section 5 or, e.g., [26]), and/or perform a simple balance adjustment between the efficiency of the compression process within the examined scheme and its cryptographic strength.

It is relatively easy to show that for the form (17) of the encryption matrix C, the maximum possible absolute value of a single element of the ciphertext matrix **Y** is equal to $\sqrt{K}\;x_{\max}$, where $x_{\max}$ is the maximum possible absolute value of the individual sample of the input image X. Based on the assumption that $x_{\max}$ is also the maximum possible value comprising the input range limit, accepted by the selected standard graphics file format, to which the user wishes to archive the encrypted image Y in the optional step (ii) of our scheme (see discussion in the first part of Section 4), we can infer that the maximum possible value of the scaling constant α used in step (i) of the analyzed scheme is $\sqrt{K}$. This inference can be summarized as:

(18) $$\max_{\begin{array}{c} i=1,\ldots ,N\\ j=1,\ldots ,M \end{array}}\left|\;{\left(\;\;Y\;\right)}_{ij}\;\right|=\sqrt{K}\;x_{\max}\;\Rightarrow \;\alpha_{\max}=\sqrt{K}.$$

As explained earlier in Section 4, choosing the value $\alpha_{\max}$ of the scaling factor α ensures that all integer projected samples $\frac{1}{\alpha_{\max}}\;Y+E$ of the scaled ciphertext $\frac{1}{\alpha_{\max}}\;Y$ will fall into an interval contained within the appropriate input range, accepted by the mentioned graphics image file format.

Let us now choose the compression transform to be 8×8 point two-dimensional discrete cosine transform of the second kind (2D-DCTII) with a matrix form (see [27]):

(19) $$A_{2D}^{\left(8\times 8\right)}=\;A_{1D}^{\left(8\right)}⊗\;A_{1D}^{\left(8\right)},$$

where the elements of 8×8 – element matrix $A_{1D}^{\left(8\right)}$ are:

(20) $$\begin{array}{c} {\left[\;A_{1D}^{\left(8\right)}\;\right]}_{\;ij}=\left\{\begin{array}{cc} \;\frac{1}{\sqrt{8}} & \mathrm{for}\;\;i=1,j=1,\ldots ,8,\\ \frac{1}{2}cos\left(\frac{\pi (i-1)(2j-1)}{16}\right) & \mathrm{for}\;\mathrm{remaining}\;\;i,j. \end{array}\right. \end{array}$$

This is an orthogonal transformation, optimal in the image compression problem for the probabilistic image model (18) with row and column autocorrelation coefficients $\rho_{r},\rho_{c}$ of the adjacent image elements equal in limit to 1, see [27].

Let us finally choose the quantization table, which is the last parameter required for the simulation model of theoretical quality characteristics of the compression process present in the examined coding scheme to be completed. Let us take the quantization table recommended in the JPEG standard (see [18], table K.1, p. 143) with the following quantization steps:

(21) $$\Delta =vec\;\;\left(\begin{array}{cccccccc} \;\;\;16 & 11 & 10 & 16 & 24 & 40 & 51 & 61\;\;\;\\ \;\;\;12 & 12 & 14 & 19 & 26 & 58 & 60 & 55\;\;\;\\ \;\;\;14 & 13 & 16 & 24 & 40 & 57 & 69 & 56\;\;\;\\ \;\;\;14 & 17 & 22 & 29 & 51 & 87 & 80 & 62\;\;\;\\ \;\;\;18 & 22 & 37 & 56 & 68 & 109 & 103 & 77\;\;\;\\ \;\;\;24 & 35 & 55 & 64 & 81 & 104 & 113 & 92\;\;\;\\ \;\;\;49 & 64 & 78 & 87 & 103 & 121 & 120 & 101\;\;\;\\ \;\;\;72 & 92 & 95 & 98 & 112 & 100 & 103 & 99\;\;\; \end{array}\right)\;.$$

The parameters $R_{x},\;C,\;A_{2D}^{\left(8\times 8\right)},\;\Delta$, and $\alpha_{\max}$ for $\sigma_{x}^{2}=1024$ and $\rho_{r}=\rho_{c}=0.95$ correspond to the simulation of the compression process, being a part of the considered scheme, which fully utilizes unmodified standard JPEG algorithm which in this case operates on W×W pixel, 8-bit grayscale image.

**Figure 5:**
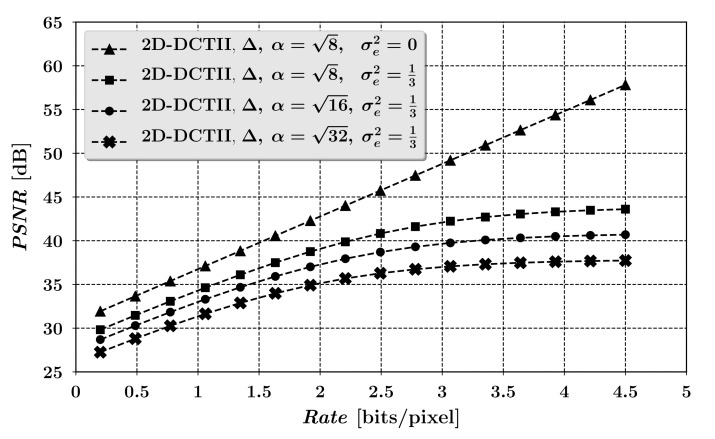
Selected quality characteristics of the image compression process.

Figure 5 shows the theoretical quality characteristics of the compression process for the examined scheme in the form of the dependence of the *PSNR* measure of image reconstruction error *D* at the output of the analyzed scheme on the minimum average bit rate *R*, resulting from Equation (14). For clarity, it is worth mentioning that in Figure 5 the $PSNR=10\log_{\;10}\left(\;255^{2}/D\;\right)$, where the mean squared error *D* is given by the relationship (14).

Additionally, to illustrate the trade-off between the scheme’s cryptographic strength, depending on the dimensions *K* of the encryption matrices $C_{i}$ (see (17) and Section 6), and its compression capabilities, three different characteristics for K=8,16 and 32 are shown in Figure 5. The last of the characteristics presented in Figure 5 applies to the case when $\sigma_{e}^{2}=0$, that is, when the user omits the optional step (ii) of the analyzed scheme in which the integer projection is performed. In such case Equation (14) does not depend on the scaling factor α, and the situation corresponds to the scheme’s implementation variant in which it is not necessary to save the intermediate encrypted image, for example, to a file in the BMP format, that is, the ciphertext Y is fed directly to the compression subsystem as a matrix of real samples. Such scenario requires only a slight modification of the standard JPEG method in such a way, that it should be acceptable to supply the JPEG’s compressor input with real sample values instead of integers.

**Figure 6:**
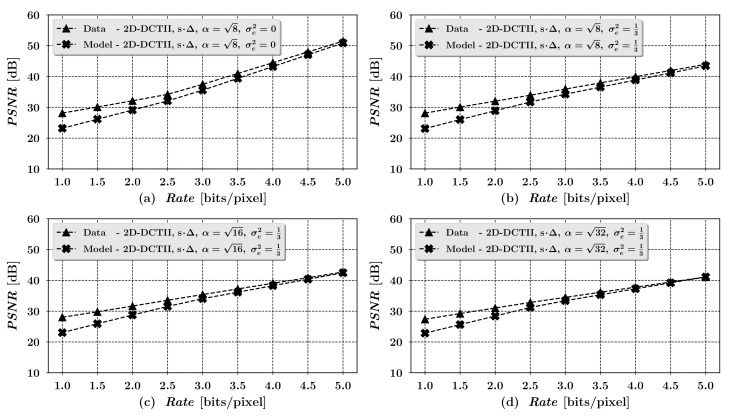
Comparison of the modeled and the sampled R(D) pairs for the ’Mandrill’ image.

Finally, it is interesting to verify, at least briefly, the accuracy of the derived high resolution approximation (14) of rate-distortion characteristics of the considered scheme on the exemplary input image. For this purpose we have chosen the 512×512-pixel, 8-bit grayscale ’Mandrill’ image (see Figure 12 in Section 7), used later in our experiments, since it enables for examining proportionally wider range of higher bit rates, in comparison to other images used in our experiments, due to its relatively noisy statistical characteristics. The image was processed exactly according to our scheme’s model (4) operation, with the use of parameters $\;C,\;A_{2D}^{\left(8\times 8\right)},\;\Delta$, and $\alpha_{\max}$ defined in Equations (17)–(21), for the block sizes K=8,16 and 32 of the encryption matrix C. To achieve variable sample bit rates for the experimental image, the quantization matrix (21) was multiplied by scaling factors $s\in R^{+}$, whose values were calculated according to a widely-used JPEG’s quantization table scaling method, described for example, in [28]. We then calculated sample bit rates for subsequent image compression qualities, determined by the consecutive values of parameter *s*, using the histogram method of differential entropy approximation for the exemplary input image, along with their corresponding sample mean squared errors of the reconstruction of the original exemplary image on the output of the analyzed scheme. Computed in such way, sample bit rates *R* and corresponding sample mean squared errors *D*, were then compared with their theoretical counterparts defined in Equations (13) and (14), calculated on the basis of the exemplary image’s sample autocovariance matrix $R_{x}$, respective quantization tables s×Δ and the integer projection error’s variance $\sigma_{e}^{2}=\frac{1}{3}$, corresponding to the integer truncation of the encrypted image data, performed in step (ii) of our simulation. The resulting graphs, showing the comparison of the modeled and the sampled rate-distortion pairs, characteristic to the examined image coding scheme for the exemplary ’Mandrill’ input image are depicted below in Figure 6.

The graphs reveal that for higher bit rates the approximate, theoretical D(R) characteristics derived for the considered scheme, match up quite accurately with their data sampled counterparts. For lower bit rates on the other hand, the derived dependencies become inaccurate, what results from the simplifications, proper for high-resolution quantization theory approximations, used in our derivations.

## 5. Fast Parametric Orthogonal Transforms

Fast parametric orthogonal transforms (FPOT) are an extension of the class of known transforms with strictly defined basis vectors (e.g., DCTII) onto the class of transforms described by the values of parameters. The parametrization allows to determine the form of the transform on the basis of the private key, or enables its automatic adaptation to a given criterion (see e.g., [29]), while maintaining fast computational structures with $O(n\log_{2}n)$ complexity, where *n* is the transform size. The fast computational structures of FPOTs can be determined in the way allowing to obtain the required properties (e.g., involutory transforms [30,31]) or can follow the fast computational structures of known orthogonal transforms. The second approach is based on the heuristic: if the known transform has good properties in solving the given class of problems, then parametrization, and the ability to adopt transform itself, can only serve to improve the results.

**Figure 7:**
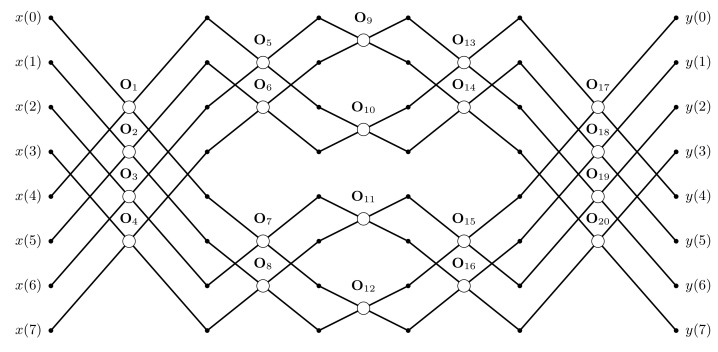
Fast structure of two-stage parametric transform for n=8.

A good example of the heuristic is FPOT with two-stage structure that follows the well known Beneš interconnection network [32] (see Figure 7, for n=8). It is well known that Beneš network is able to realize any permutation in the set of *n* elements. Hence, its computational structure can be effective from the point of view of data encryption. The parametrization of Beneš network involves the use of base operations (described symbolically as ’∘’) for example, defined as:

(22) $$\begin{array}{c} O_{i}\left(\alpha_{i}\right)=\left[\begin{array}{cc} \cos\alpha_{i} & \sin\alpha_{i}\\ -\sin\alpha_{i} & \cos\alpha_{i} \end{array}\right], \end{array}$$

where $\alpha_{i}$ is the operator’s parameter, and *i* is an index of the operator with $i=1,\cdots ,L_{P}\left(n\right)$, while $L_{P}\left(n\right)$ describes the total number of transform parameters. For the two-stage structure we have $L_{P}\left(n\right)=\frac{n}{2}\left(2\log_{2}n-1\right)$. And then $\left\{\alpha_{i}:i=1,2,\cdots ,L_{P}\left(n\right)\right\}$ is the set of parameters whose values fully define the form of the resulting transform.

In the task of encryption-then-compression of natural images FPOTs can be used at the image encryption stage to implement the block elements of the encryption matrix C, and also realize all the necessary permutations (see Section 3). In the first case we can use the structure shown in Figure 7 with base operations in the form of rotations defined as (22). Then the mapping of the bits of the private key (*q* bits resulting in $\xi =2^{q}$ different values) onto the values of parameters (from the range [0,2π)) can be implemented according to the formula $\alpha_{i}=2\pi d_{i}\xi^{-1}$, where $d_{i}\in \left\{0,1,\cdots ,\xi -1\right\}$ for $i=1,2,\cdots ,L_{P}\left(n\right)$ (c.f. [13,31]). Then the concatenation of the bit representations of numbers $d_{i}$ is the part of the private key K describing the transformation.

In the case of permutations the Beneš network (see Figure 7) in its direct form can be used. Here, however, the base operations can be reduced to simple variants $T_{i}$ changing the order of elements if the $s_{i}$ parameter equals 1, that is,:

(23) $$\begin{array}{c} T_{i}\left(s_{i}\right)=\left[\begin{array}{cc} 1-s_{i} & s_{i}\\ s_{i} & 1-s_{i} \end{array}\right], \end{array}$$

where $s_{i}\in \left\{0,1\right\}$. Then any permutation in the *n*-element set can be described by the sequence $\left\{s_{i}:i=1,2,\cdots ,L_{P}\left(n\right)\right\}$ of binary numbers, whose concatenation will complement the private key K.

## 6. Analysis of the Encryption Efficiency of the Considered Scheme

An analysis of encryption efficiency is an essential step in the design of any cryptographic system. In the case of the analyzed approach, such an analysis was performed: (i) based on the known measures used to evaluate the effectiveness of image cryptographic methods, that is, combinatorial analysis and quality indicators in the form of Histogram Analysis (HA), Maximum Deviation (MD), Correlation Coefficient (CC), or Irregular Deviation (ID) (see [33,34]), (ii) as a statistical analysis aimed at deriving dependencies allowing to determine the probability of obtaining a reconstruction error at a level not greater than a given value in the case of an attempt to randomly guess the proper encryption key.

### 6.1. Combinatorial Analysis

The combinatorial analysis applies to the considered encryption scheme (see Section 3), where the encryption matrix C is a composite of permutation matrices $P_{r}$, $P_{c}$ (M×M element matrices) and a block-diagonal matrix with K×K element blocks $C_{l}$ for l=1,2,⋯,M/K. We assume that both permutation matrices and the element mixing matrices $C_{l}$ are implemented with use of fast parametric transforms modeled as the two-stage structure of the Beneš network (see Section 4). In case of Beneš network the number of free parameters equals $L_{P}\left(n\right)=\frac{n}{2}\left(2\log_{2}n-1\right)$, where *n* is the size of the transform. It is assumed that for $C_{l}$ block matrices with base operations of the form (22) that each of the $\alpha_{i}$ parameters for $i=1,2,\cdots ,L_{P}\left(n\right)$ is quantized to a number of $\xi =2^{q}$ values $\alpha_{i}=2\pi d_{i}\xi^{-1}$, which are evenly distributed over the range [0,2π) with $d_{i}\in \left\{1,2,\cdots ,\xi \right\}$ being an integer that describes the value of the rotation angle in terms of *q* bits in a natural binary representation. The Beneš network with base operations of the form (23) performs any permutation in the *m*-element set, where *m* is the transform size, and each $s_{i}$ parameter is a one-bit integer, that is, $s_{i}\in \left\{0,1\right\}$. Thus, the length of the private key expressed in bits, which is the concatenation of binary representations of $d_{i}$ and $s_{i}$ parameters, can be determined according to the formula $L_{K}\left(K,M,q\right)=q\left(\frac{M}{K}\right)L_{P}\left(K\right)+2L_{P}\left(M\right)$, where M/K is the number of $C_{l}$ block matrices.

In case of an attack consisting in guessing a private key for the decryption step, the probability of drawing a key that differs by the number of κ bits in terms of the Hamming distance from the key used at the encryption stage can be described using the Bernoulli distribution with the probability of success equal to $p=\frac{1}{2}$. The expected value of κ variable, that is, the average number of bits distinguishing both keys, will then be equal to half the key length, that is, $E\left\{\kappa \right\}=\frac{1}{2}L_{K}\left(K,M,q\right)$. In turn, the probability of drawing a key that differs by the number of $\kappa_{0}$ bits can be described as:

P{κ=κ0}=LK(K,M,q)κ0×2−LK(K,M,q).

For example, with image of the size 512×512 pixels, which was divided into fragments consisting of 8×8 pixels, and the size of the $C_{l}$ block matrices was assumed to be the size of vectors $X_{k}$, we get K=64 and M=4096. Assuming the value of q=4 bits, the resulting length of the private key will be equal to $L_{K}\left(64,4096,4\right)=256L_{P}\left(64\right)+2L_{P}\left(4096\right)=$ 184,320 bits. Thus, the probability of guessing the encryption key will be approximately $10^{-55,485}$. For a key that differs by one bit from the encryption key we have $0.38\times 10^{-55,479}$, and for a key that differs by two bits $0.41\times 10^{-55,474}$. For $\kappa_{0}=$46,080 (i.e., for 25% of the key length) the probability will be $0.11\times 10^{-10,473}$. The obtained exemplary values of probabilities are negligibly small, even for relatively small size of $C_{l}$ block matrices, which is K=64. On this basis, we can conclude that the examined encryption method in conjunction with the used scheme of construction of matrix C can be characterized by high combinatorial complexity. This complexity can be further increased by increasing the value of the *K* parameter.

### 6.2. Statistical Analysis of the Decryption Error

Let there be a given set O(m) (not necessarily understood in the sense of a consistent algebraic structure) of random orthogonal matrices with the dimension m×m elements (i.e., if only we have A∈O(m) then $AA^{T}=A^{T}A=I$, where I is an identity matrix). By $a_{ij}$ for i,j=1,2,⋯,m we denote the elements of matrix A∈O(m), which are also random variables. We further assume that the matrices belonging to the set O(m) have the following properties: (i) *property of zero expected value*: expected values of random variables $a_{ij}$ for i,j=1,2,⋯,m are zero, that is, $E\{a_{ij}\}=0$ holds; (ii) *stationarity property*: variances of $a_{ij}$ random variables are constant and equal to $m^{-1}$, that is, $E\left\{a_{ij}^{2}\right\}=m^{-1}$ for i,j=1,2,⋯,m; (iii) *lack of correlation between matrix elements*: for any two different random variables $E\{a_{ij}a_{kl}\}=0$ is true, where i,j,k,l=1,2,⋯,m, and i≠k, and j≠l. In practice, matrices from the set O(m), which have the mentioned properties, can be generated: as the result of Gram-Schmidt orthogonalization of random matrices, as a product of random Housholder’s transformation or Given’s rotation matrices, or with use of the fast orthogonal parametric transforms.

Let X be an N×M element matrix representing input data arranged as a set of *M* image blocks expanded into *N*-element column vectors (see Sec. III). Input data, known as the plain text, is encrypted using the encryption matrix $C_{I}\in O\left(M\right)$, and the encryption process can be described as:

$$\begin{array}{c} Y=XC_{I}, \end{array}$$

where Y is N×M element resulting matrix, that is, the ciphertext. The column vectors of this matrix are the encrypted forms of the column plain text vectors. The decryption process follows the following formula:

$$\begin{array}{c} \bar{X}=YC_{\mathrm{II}}, \end{array}$$

where $C_{\mathrm{II}}\in O\left(M\right)$ is the decryption matrix. If $C_{\mathrm{II}}\neq C_{I}^{T}$, then also $\bar{X}\neq X$, and the absolute error of the signal reconstruction can be defined using the Hilbert-Schmidt operator as:

(24) $$\begin{array}{c} D=tr\{{\left(X-\bar{X}\right)}^{T}\left(X-\bar{X}\right)\}, \end{array}$$

where tr{×} is the trace of a matrix and ${\left(\times \right)}^{T}$ describes matrix transposition. The relative value of *D* error related to the energy of the input signal can be defined as:

(25) $$\begin{array}{c} D_{*}=D/tr\left\{X^{T}X\right\}. \end{array}$$

Next substituting $\bar{X}$ in formula (24) with $\bar{X}=XC_{I}C_{\mathrm{II}}$ we obtain after elementary matrix transformations the formula:

(26) $$\begin{array}{c} D=2tr\{\left(I-C_{I}C_{\mathrm{II}}\right)X^{T}X\}, \end{array}$$

where I is M×M element identity matrix. In the rest of the section we will calculate the expected value, variance, and the statistical distribution of the reconstruction error calculated for randomly selected decryption matrices $C_{\mathrm{II}}\in O\left(M\right)$.

The expected value of the reconstruction error (24) calculated for randomly selected matrices $C_{\mathrm{II}}$ can be expressed as:

(27) $$\begin{array}{c} \bar{D}=E\left\{2tr\{\left(I-C_{I}C_{\mathrm{II}}\right)X^{T}X)\}\right\}=2tr\left\{\left(I-C_{I}E\left\{C_{\mathrm{II}}\right\}\right)X^{T}X\right\}. \end{array}$$

Taking into account the assumed statistical properties of matrices from the set O(M), that is, $E\{C_{\mathrm{II}}\}=O$, where O is the null matrix, we can write:

(28) $$\begin{array}{c} \bar{D}=2tr\left\{X^{T}X\right\}. \end{array}$$

The result in (28) gives immediately $D_{*}=2$, which means that the expected value of the relative reconstruction error is twice the energy of input signal. In terms of the known measure of signal reconstruction quality, *Signal to Noise Ratio* (SNR), this corresponds approximately to −3 dB.

The variance of the absolute error value can be described using the following relationship:

$$\begin{array}{c} \sigma_{D}^{2}=E\left\{{\left(D-\bar{D}\right)}^{2}\right\}, \end{array}$$

where the expected value is calculated relative to the matrix $C_{\mathrm{II}}\in O\left(M\right)$. Next taking into account the expression (26) and the expected value $\bar{D}$ (see (28)), we get the formula:

(29) $$\begin{array}{c} \sigma_{D}^{2}=4E\left\{tr{\left\{C_{\mathrm{II}}X^{T}Y\right\}}^{2}\right\}. \end{array}$$

Having in mind previously formulated properties of stationarity and the lack of correlation of elements of matrices from the set O(m), we can rewrite (29) in a simpler form:

(30) $$\begin{array}{c} \sigma_{D}^{2}=\frac{4}{M}tr\left\{X^{T}YY^{T}X\right\}. \end{array}$$

We should note that formula (30) does not depend on the encryption matrix $C_{I}$, since $tr\left\{X^{T}YY^{T}X\right\}=tr\left\{X^{T}XX^{T}X\right\}$. It can be also proved that $tr\left\{X^{T}XX^{T}X\right\}\leq tr{\left\{X^{T}X\right\}}^{2}$. This allows to upper bound the variance of absolute error as:

(31) $$\begin{array}{c} \sigma_{D}^{2}\leq \frac{4}{M}tr{\left\{X^{T}X\right\}}^{2}. \end{array}$$

In case of relative error we obtain an analogical estimate:

(32) $$\begin{array}{c} \sigma_{D_{*}}^{2}\leq \frac{4}{M}. \end{array}$$

**Figure 8:**
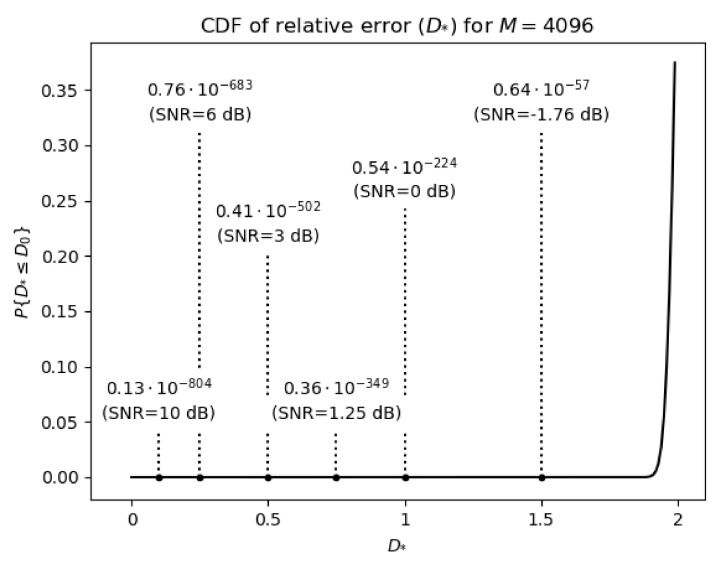
The values of the probability of obtaining the relative error at the level not greater than $D_{0}$ for M=4096 and the quality indicators of the image reconstruction in the form of Signal to Noise Ratio (SNR) corresponding to the selected values of $D_{0}$.

**Figure 9:**
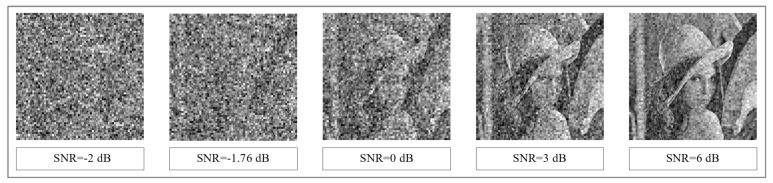
Sample results of decryption of ’Lena’ image for different values of SNR measure

The statistical distribution of the value of the absolute error *D*, and also the relative error $D_{*}$, is the normal distribution. It results directly from the fact that the value of *D* is created as a weighted sum of random variables with zero expected value, that is, the elements $c_{ij}$ for i,j=1,2,⋯,M of matrix C, and the validity of this statement can be proved on the basis of the Central Limit Theorem. Hence, the probability density function of *D* can be fully characterized by its expected value $\bar{D}$ and the variance $\sigma_{D}^{2}$. Similarly ${\bar{D}}_{*}$ and $\sigma_{D_{*}}^{2}$ fully define the distribution of relative error. This allows for simple estimation of the probability of obtaining an error value not greater than a given value. For example, in case of $D_{*}$ the probability of getting an error value not greater than $D_{0}$ is described as:

(33) $$\begin{array}{c} P\left\{D_{*}\leq D_{0}\right\}=\frac{1}{2}\left(1+\mathrm{erf}\left(\frac{D_{0}-{\bar{D}}_{*}}{\sigma_{D_{*}}\sqrt{2}}\right)\right), \end{array}$$

where we assume ${\bar{D}}_{*}=2$ and $\sigma_{D_{*}}=2/\sqrt{M}$. An exemplary results for ’Lena’ image with resolution 512×512, and image blocks of sizes 8×8 pixels, which correspond to N=64 and M=4096, are presented in Figure 8 for the following values of $D_{0}=\left\{0.1,0.25,0.5,0.75,1.0,1.5\right\}$. The obtained results are plotted as the cumulative probability density (CDF) function of the $D_{*}$ random variable. It should be noted that for images, unlike text data, changing one or even several bits of binary representation of pixel luminance does not mean that the image content will be unreadable. However, the SNR quality measure allows to assess the legibility of the image after decryption. Figure 9 shows how the legibility of ’Lena’ image changes in the function of SNR measure. An analysis of results shows that the values of SNR measure close to −2 dB guarantee good hiding of image content. On the basis of formula (32) the probability of obtaining SNR values not lower than −2 dB (i.e., for $D_{0}\approx 1.6$) can be determined. This probability will be around $0.18\times 10^{-40}$. The probabilities for the remaining values of SNR are shown in Figure 8.

### 6.3. Analysis Based on the Histogram

**Figure 10:**
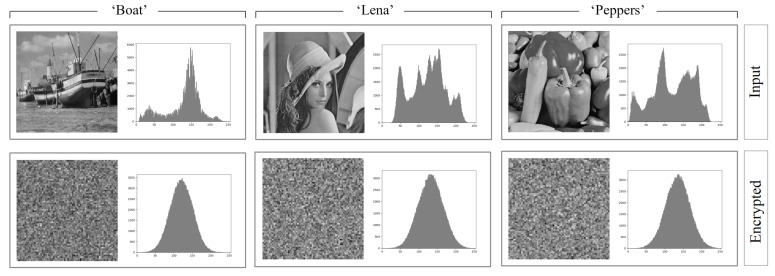
Results of the histogram analysis for the analyzed method and sample natural images (’Boat’, ’Lena’ and ’Peppers’).

From the point of view of statistical analysis the histogram describes the probability distribution of the luminance values of individual pixels in the image (grayscale images). In case of encryption algorithms the algorithm having good properties is the one that produces an even distribution of symbols representing the data after encryption [34]. The even distribution corresponds to the same frequency or probability of occurrence of particular symbols in the ciphertext (pixel luminance values). Since the considered method is based on matrix multiplication, that is, it is a linear technique, and the pixel values in the image obtained after encryption are formed as the weighted sums (with the elements of the encryption matrix) of luminance values of input image pixels, then, according to the Central Limit Theorem, the resulting distribution will be a normal distribution (or will be close to such distribution). This is an inherent feature of linear methods. Figure 10 shows histograms for three sample images calculated before and after encryption using the examined method. The luminance probability distribution of the pixels after encryption looks in a statistical sense like normally distributed noise.

### 6.4. Maximum Deviation Index

The Maximum Deviation (MD) index allows to evaluate the efficiency of the encryption process in terms of the deviation calculated between the distributions of pixel luminance values in the input and encrypted images. In order to do this, first the histograms of both images should be calculated, that is, $H_{I}\left(i\right)$ for the input image and $H_{C}\left(i\right)$ for the encrypted image, where i=0,1,⋯,255. Then the value of the deviation index can be determined on the basis of the following formula:

$$\begin{array}{c} \eta_{MD}\left(H_{D}\right)=\left(\frac{H_{D}\left(0\right)+H_{D}\left(255\right)}{2}\right)+\sum_{i=1}^{254}H_{D}\left(i\right), \end{array}$$

where $H_{D}\left(i\right)=\left|H_{I}\left(i\right)-H_{C}\left(i\right)\right|$. The greater the value of $\eta_{MD}$, the more the encrypted image differs from the input.

Table 1 contains MD index values obtained for three sample images (’Boat’, ’Lena’ and ’Peppers’) with four encryption algorithms, that is, method from paper [9], the considered method, and two popular symmetric block ciphers, that is, Advanced Encryption Standard (AES) and Data Encryption Standard (DES) (see [35]). In case of block ciphers, we used the Cipher Block Chaining (CBC) scheme, which consists in sequential binding of encrypted blocks, and allows to avoid the repetition of some patterns in the encrypted image. The obtained results are comparable in terms of an order of magnitude. The results obtained with method from [9] and the considered method are very close. For images ’Boat’ and ’Lena’ the method from paper [9] allows to obtain better results, while for ’Peppers’ image the considered method generates the highest value of $\eta_{MD}$ index. The results obtained with AES and DES ciphers are very similar. It is possible to indicate images for which block ciphers give higher values of $\eta_{MD}$ index than the examined method (’Boat’, ’Lena’). However, we can also indicate images for which the opposite relationship holds (’Peppers’). It should be noted that $\eta_{MD}$ index allows to assess the quality of the encryption method based only the notion of the pixel luminance distribution.

### 6.5. Correlation Coefficient Index

By the Correlation Coefficient (CC) index $\eta_{CC}$, we understand the correlation calculated between the input and the encrypted image. Obviously $\eta_{CC}\in \left[-1,1\right]$. From the viewpoint of encryption task the desired value of $\eta_{CC}$ is zero, which corresponds to the lack of statistical similarity between both images. Let U with dimensions W×H represent the input image and V the encrypted image. Then the $\eta_{CC}$ index can be determined on the basis of the following formula:

$$\begin{array}{c} \eta_{CC}\left(U,V\right)=\frac{\left(\sum_{i=1}^{W}\sum_{j=1}^{H}\left(u_{ij}-\mu \left(U\right)\right)\left(v_{ij}-\mu \left(V\right)\right)\right)}{WH(\sigma (U)\sigma (V))}, \end{array}$$

where $u_{ij}$ and $v_{ij}$ for i=1,2,⋯,W, j=1,2,⋯,H are the pixel luminance values of U and V images, while μ(Z) and σ(Z) are the mean value and the standard deviation of the pixel luminance values, respectively, that is,:

$$\begin{array}{c} \mu \left(Z\right)=\frac{1}{WH}\left(\sum_{i=1}^{W}\sum_{j=1}^{H}z_{ij}\right), \end{array}$$

in case of the mean value and for standard deviation:

$$\begin{array}{c} \sigma \left(Z\right)=\sqrt{\frac{1}{WH}\left(\sum_{i=1}^{W}\sum_{j=1}^{H}{\left(z_{ij}-\mu \left(Z\right)\right)}^{2}\right)}. \end{array}$$

The results of the experimental measurements of $\mu_{CC}$ index for sample images (’Boat’, ’Lena’ and ’Peppers’) obtained for the method from paper [9], the considered encryption method, and both AES and DES block ciphers, are presented in the Table 2.

Based on the analysis of results, we can conclude that $\mu_{CC}$ index values for the considered method are close to zero. The method proposed in [9] allowed to obtain a better result for ’Boat’ image. The results obtained for block ciphers are on average an one order of magnitude lower than the results obtained with the methods dedicated to image encryption.

### 6.6. Irregular Deviation Index

The Irregular Deviation (ID) index is based on measuring the deviation of pixel values in encrypted image relative to pixel values in an input image. Let matrices U and V (W×H element matrices) describe pixel luminances in input and encrypted images, respectively. In order to determine the value of the ID index ($\mu_{ID}$), first we have to calculate the matrix T, which holds the modules of differences between the elements of matrices U and V, that is, $t_{ij}=\left|u_{ij}-v_{ij}\right|$ for i=1,2,⋯,W and j=1,2,⋯,H. Then, it is required to determine the $H_{T}$ histogram of occurrences of individual elements in matrix T. The next step is to count the average number of pixels, which for each value of luminance differed from the input value. It can be done on the basis of $H_{T}$ histogram using the formula:

$$\begin{array}{c} \mu \left(H_{T}\right)=\frac{1}{256}\sum_{i=0}^{255}H_{T}\left(i\right), \end{array}$$

where $H_{T}\left(i\right)$ for i=0,1,⋯,255 are the individual values of $H_{T}$ histogram. The mean value $\mu (H_{T})$ describes the model histogram, which in optimal case should take form of the uniform distribution. Then, on the basis of $H_{T}$ and $\mu (H_{T})$, we build the ${\bar{H}}_{T}$ histogram, which in turn describes the deviation of the $H_{T}$ from the optimal uniform distribution. In this way we obtain ${\bar{H}}_{T}\left(i\right)=\left|H_{T}\left(i\right)-\mu \left(H_{T}\right)\right|$ for i=0,1,⋯,255. Based on the ${\bar{H}}_{T}$ histogram, we can directly calculate the value of the $\mu_{ID}$ index as the area under the histogram ${\bar{H}}_{T}$:

$$\begin{array}{c} \mu_{ID}\left({\bar{H}}_{T}\right)=\sum_{i=0}^{255}{\bar{H}}_{T}\left(i\right). \end{array}$$

The smaller the value of $\mu_{ID}$ index, the greater the efficiency of the encryption method. Table 3 contains sample values of $\mu_{ID}$ index calculated for exemplary natural images (’Boat’, ’Lena’, ’Peppers’) and method from paper [9], the considered encryption method, and AES and DES block ciphers.

The analysis of the experimental results shows that the considered encryption method can be characterized by higher values of the $\mu_{ID}$ index, on average by 29%, compared to the results for AES and DES block ciphers. In turn, the results obtained with block ciphers are comparable. The method proposed in paper [9] gives results close to the results obtained with the considered method, and better for ’Lena’ and ’Peppers’ images.

## 7. Experimental Results in Efficiency of the Compression Process

**Figure 11:**
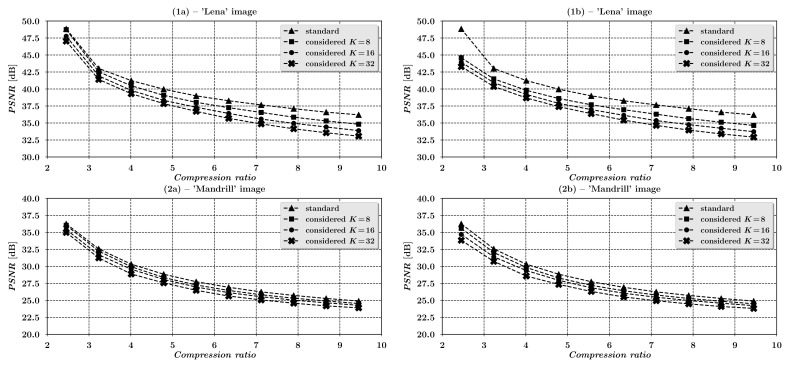
Graphs of dependences of PSNR image reconstruction quality measures on the compression ratios for the standard and the considered method, (**1a**,**2a**)—Lena’ and ’Mandrill’ images without integer projection, (**1b**,**2b**)—’Lena’ and ’Mandrill’ images with integer projection.

**Figure 12:**
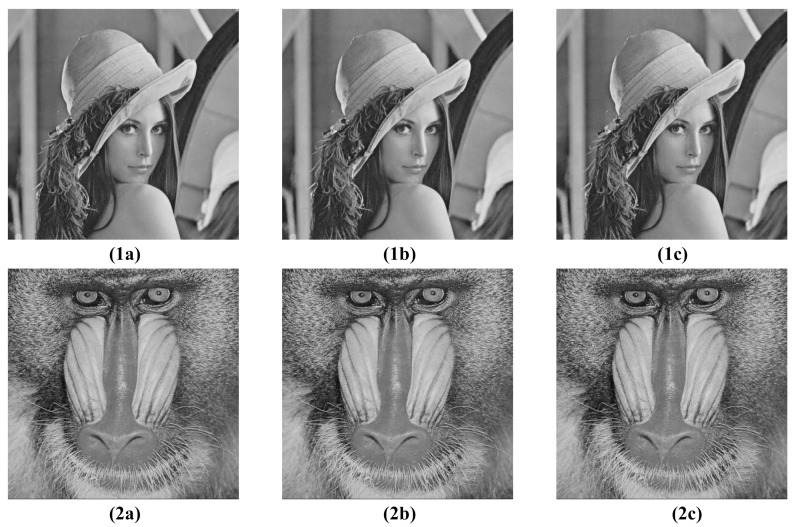
Visual comparisons of image reconstruction quality for standard and the considered method with integer projection in step (ii) of the examined scheme, (**1a**)—original image ’Lena’, (**1b**)—compression with standard JPEG method, (**1c**)—compression with the considered method, compression coeff. ≈5, (**2a**)—original image ’Mandrill’, (**2b**)—compression with standard JPEG method, (**2c**)—compression with the considered method, compression coeff. ≈5.

In order to verify the effectiveness of compression a series of tests were performed on natural images with different statistical characteristics. In Table 4 and in the graphs in Figure 11, the results for two standard, representative test images are shown, that is, the ’Lena’ image (1a) and the ’Mandrill’ image (2a) in Figure 12. Both images are 8–bit grayscale with resolutions of 512×512 pixels. The images were compressed with the standard JPEG method and, for comparison, encrypted and compressed with the analyzed scheme. At the compression stage of the considered coding scheme the standard JPEG algorithm was applied in two variants. In the first one, it was utilized without any modification to its original form and operated on ciphertext previously projected, by truncation towards zero, to their respective integers. Such projection corresponds to the situation in which the user saves the encrypted image to one of the standard graphics formats, for example, BMP format, before it is actually compressed. In the second variant the projection was not performed and the input of the JPEG compressor was supplied with real values obtained after encryption of the input image. This required only a slight modification of the JPEG method, by allowing the input of the compressor to be fed with real samples instead of integers. Moreover, in order to examine the influence of the choice of dimensions *K* of encryption matrices $C_{i}$, for each of the two described variants of the examined method, the effectiveness of the compression process was evaluated for three different values of *K*, namely for K=8,16 and 32. For all the described tests the compression levels were regulated by appropriate modification [36], p. 122 of the standard quantization table, given in (21). The PSNR was used as the image reconstruction quality measure, calculated as:

$$\mathrm{PSNR}=10\times \log_{\;10}\;\left[\;\;\;\;\frac{255^{\;2}W^{2}}{\sum_{i=\;1}^{W}\sum_{j=\;1}^{W}{\left(\;U_{ij}-{\hat{U}}_{ij}\right)}^{2}\;}\;\;\;\right]\;,$$

where W=512 stands the vertical and horizontal resolutions of images, $U_{ij}$ and ${\hat{U}}_{ij}$, i,j=1,…,W stand for the original and reconstructed images, respectively, for each of the tested methods. Results of all the experiments are shown in Table 4.

## 8. Summary and Conclusions

The paper analyzes an image encryption scheme that preserves input data statistics and can be used in conjunction with a popular JPEG image compression standard. In this way the considered encryption method together with JPEG standard constitute a highly efficient realization of encrypt-then-compress image processing framework. Moreover, thanks to the use of fast parametric linear transforms the presented scheme is computationally efficient and does not alter the statistical characteristics of input images what allows to preserve the effectiveness of the compression process. The obtained results indicate that for a wide range of compression ratios the effectiveness of compression process, understood in the terms of the quality of reconstructed images evaluated with PSNR measure, in case of compressing encrypted images is comparable to the respective effectiveness of the standard JPEG method alone, that is, applied without the encryption stage. It should be noted that the maximum quality differences of the reconstructed images in relation to the standard JPEG algorithm (for the practical case with K=8, the compression ratio ≈5 and the use of the unmodified JPEG method, with the encrypted image data saved to an intermediate graphics file, for example, BMP format file) were 1.31 dB and 0.53 dB for the test images ’Lena’ and ’Mandrill’, respectively, what can be considered to be a highly satisfactory result, consistent with theoretical predictions presented in Section 4 and confirmed by visual comparisons depicted in Figure 12.

This paper also presents the detailed analysis of the efficiency of the encryption step using common approaches, that is, histogram analysis, Maximum Deviation (MD), Correlation Coefficient (CC), Irregular Deviation (ID), or combinatorial and statistical analysis. The analyzed method is also compared in terms of the mentioned encryption efficiency measures (i.e., MD, CC and ID) to the method from paper [9], and symmetric block ciphers DES and AES. The choice of the method from [9] is due to its comparable performance in compression of encrypted images, and to the fact that the method is dedicated for encryption and compression of natural images. The analysis of experimental results shows that the efficiencies of the encryption steps of the analyzed method and the method from paper [9] are very close. Moreover, the results obtained for the considered method with MD, CC and ID indexes are comparable to those for symmetric block ciphers DES and AES, although in most cases worse, except for the MD index in the case of the ’Peppers’ image. Furthermore, the histogram analysis shows that the pixel intensity distribution of encrypted images is approximately normal. The statistical analysis of the encryption stage indicates high combinatorial complexity of the examined method, since the number of bits of the private key is linear-logarithmically dependent on the block size of the encryption transform matrix, what for the block size N=64, number of blocks M=4096 and the number of 4 bits per single key parameter gives a total length of a key $L_{K}\left(64,4096,4\right)=$ 184,320 bits. This also guarantees a low probability of guessing a private key, which makes possible to decrypt images with a PSNR value greater than the certain threshold allowing for recognition of decrypted images. For example, for PSNR not greater than −1.76 dB the mentioned probability is equal to $0.64\times 10^{-57}$.

The experimental results along with the presented theoretical analysis show that the considered scheme is highly efficient in terms of its encryption capabilities and compression quality, as well as in terms of ease of its application in conjunction with JPEG image compression standard. It should be noted that the analyzed encryption method preserves the image statistics in the form of the auto-correlation matrix. As so, it can be used in connection with any image compression method based on the form of the auto-correlation matrix.

## Figures and Tables

**Table 1:** The values of $\eta_{MD}$ index for sample images (’Boat’, ’Lena’, ’Peppers’) and various encryption methods (considered, AES and DES block ciphers in the CBC scheme, method from paper [9]).

Image	Method [9]	Considered	AES + CBC	DES + CBC
’Boat’	169,848.5	161,842.5	221,312	221,746.5
’Lena’	133,883	126,087	169,378.5	168,856.5
’Peppers’	133,755	192,357.5	144,174	144,084.5

**Table 2:** The values of the $\eta_{CC}$ index for sample images (’Boat’, ’Lena’, ’Peppers’) and selected encryption methods (Considered, Advanced Encryption Standard (AES) and Data Encryption Standard (DES) block ciphers in Cipher Block Chaining (CBC) scheme, method from [9]).

Image	Method [9]	Considered	AES + CBC	DES + CBC
’Boat’	$-0.53\times 10^{-3}$	$-0.17\times 10^{-1}$	$-0.18\times 10^{-2}$	$-0.47\times 10^{-2}$
’Lena’	$-0.31\times 10^{-1}$	$0.10\times 10^{-1}$	$0.45\times 10^{-2}$	$-0.95\times 10^{-3}$
’Peppers’	$0.47\times 10^{-1}$	$-0.50\times 10^{-2}$	$0.14\times 10^{-2}$	$-0.13\times 10^{-3}$

**Table 3:** The values of $\mu_{ID}$ index for sample images (’Boat’, ’Lena’, ’Peppers’) and selected encryption methods (considered, AES and DES block ciphers in CBC schema, method from [9]).

Image	Method [9]	Considered	AES + CBC	DES + CBC
’Boat’	250,726	248,506	186,588	186,652
’Lena’	219,236	233,898	180,002	180,992
’Peppers’	243,636	247,716	166,458	166,104

**Table 4:** Dependence of Peak Signal-to-Noise Ratio (PSNR) image reconstruction quality on the compression ratios for the standard and the considered method.

Compression Coefficient	PSNR Values [dB]													
	’Lena’ Image							’Mandrill’ Image						
	Standard Method	Considered Method						Standard Method	Considered Method					
		Without Integer Projection			With Integer Projection				Without Integer Projection			With Integer Projection		
		K=8	K=16	K=32	K=8	K=16	K=32		K=8	K=16	K=32	K=8	K=16	K=32
2.455	48.839	48.726	47.758	47.032	44.589	43.935	43.288	36.250	36.032	35.406	34.981	35.582	34.697	33.829
3.232	43.020	42.524	41.922	41.401	41.488	40.934	40.377	32.567	32.236	31.699	31.241	32.039	31.396	30.734
4.009	41.240	40.479	39.797	39.311	39.810	39.205	38.687	30.314	29.944	29.575	28.878	29.831	29.385	28.577
4.787	39.967	39.071	38.328	37.846	38.597	37.883	37.404	28.841	28.356	28.069	27.568	28.281	27.941	27.341
5.564	38.989	38.041	37.316	36.714	37.676	36.971	36.371	27.737	27.260	26.955	26.472	27.208	26.850	26.304
6.341	38.271	37.254	36.406	35.682	36.953	36.128	35.421	26.916	26.450	26.128	25.632	26.403	26.039	25.497
7.118	37.660	36.550	35.577	34.865	36.293	35.353	34.648	26.242	25.820	25.529	25.055	25.783	25.454	24.938
7.896	37.104	35.856	34.949	34.148	35.641	34.756	33.956	25.720	25.315	25.029	24.577	25.274	24.972	24.476
8.673	36.595	35.303	34.416	33.564	35.116	34.230	33.393	25.275	24.900	24.631	24.181	24.863	24.579	24.092
9.450	36.201	34.818	33.876	33.067	34.642	33.723	32.921	24.891	24.530	24.270	23.893	24.496	24.221	23.809

## Data Availability

Not applicable.
